# Comparative analysis of mitochondrial DNA datasets indicates that *Toxascaris leonina* represents a species complex

**DOI:** 10.1186/s13071-019-3447-2

**Published:** 2019-05-02

**Authors:** Yuan-Chun Jin, Xiang-Yong Li, Jin-Hui Liu, Xing-Quan Zhu, Guo-Hua Liu

**Affiliations:** 1 Hunan Provincial Key Laboratory of Protein Engineering in Animal Vaccines, College of Veterinary Medicine, Hunan Agricultural University, Changsha, 410128 Hunan People’s Republic of China; 2 Changsha Ecological Zoo, Changsha, 410118 Hunan China; 3 Hunan Co-Innovation Center of Animal Production Safety, Changsha, 410128 Hunan People’s Republic of China; 4 State Key Laboratory of Veterinary Etiological Biology, Key Laboratory of Veterinary Parasitology of Gansu Province, Lanzhou Veterinary Research Institute, Chinese Academy of Agricultural Sciences, Lanzhou, 730046 Gansu People’s Republic of China

**Keywords:** Nematode, *Toxascaris leonina*, Species complex, Cheetah, Mitochondrial genome, Phylogenetic analyses

## Abstract

**Background:**

*Toxascaris leonina* is one of the most common intestinal parasites of canids and felids. In this study, we characterised the entire mitochondrial (mt) genome sequence of *T. leonina* from the cheetah and compared it with that of *T. leonina* from the dog.

**Results:**

The entire mt genome sequence of *T. leonina* from the cheetah is 14,685 bp in size, which is 375 bp longer than that from the dog, and it is 408 bp longer than that from the South China tiger. The overall nucleotide sequence (except for the non-coding region) identity was 92.8% between the two mt genomes of *T. leonina* from the cheetah and the dog. For the 12 protein-coding genes, sequence difference between *T. leonina* from the cheetah and the dog was 5.0–9.7% at the nucleotide level and 1.0–7.2% at the amino acid level. Moreover, comparison of mt *cox*1 sequences among *T. leonina* isolates (*n* = 23) from different hosts revealed substantial nucleotide differences (10.6%). Phylogenetic analysis showed the separation of *T. leonina* from canid and felid hosts into three distinct clades.

**Conclusions:**

Taken together, these mtDNA datasets indicate that *T. leonina* from canid and felid hosts represents a species complex. Our results have implications for further studies of the molecular epidemiology, systematics and population genetics of this nematode.

**Electronic supplementary material:**

The online version of this article (10.1186/s13071-019-3447-2) contains supplementary material, which is available to authorized users.

## Background

Nematodes (roundworms) are common parasites which inhabit in the gastrointestinal tract of humans, and domestic and wild animals, and most of them can cause significant economic losses and public health problems [1]. *Toxascaris leonina* (Nematoda: Ascarididae) is a common nematode of various animals, including dogs, cats, wolves, tigers, lions and foxes. Although it has a diverse range of definitive hosts, to date, it has been the only species described in the genus *Toxascaris*. Adult *T. leonina* parasitize in the small intestines of the definitive hosts and can cause serious disease in young animals [2]. Importantly, larvae of *T. leonina* can also infect humans, posing a potential public health problem [3].

The species identification of parasites is of significance for studying epidemiology, systematics, diagnostics and population genetics of parasites [4]. Usually, nematodes are identified based on morphological features, hosts, and geographical distributions. However, it may not always be possible to identify to the species level nematodes from different hosts and geographical locations based on these criteria. To overcome the limitation of morphological taxonomy, molecular/DNA markers have been extensively used for species identification and differentiation of various nematodes [5–7]. Recently, our preliminary comparative study has indicated that the mitochondrial (mt) *cox*1 gene sequence of *T. leonina* from cheetahs and dogs differed by 6.8% [8]. In addition, substantial nucleotide difference in part of the mt *nad*1 (9.0%) and *nad*4 (10.8%) were also detected between *T. leonina* from canid and felid hosts [9]. These findings raised the possibility that *T. leonina* may represent a species complex. However, this hypothesis was proposed based on a small molecular dataset. Therefore, this hypothesis should be tested by using more datasets. Mt genome (containing 36–37 genes) has been considered a suitable marker for the species identification and differentiation of many nematodes [10]. The mt genomes of *T. leonina* from canids (such as the dog) and felids (such as the South China tiger) have been sequenced and characterised previously [11, 12], but the *T. leonina* mt genome sequence from the South China tiger has not yet been deposited in the GenBank NCBI database. Moreover, it is yet unknown whether there are significant differences in the entire mt nucleotide and amino acid sequences between *T. leonina* from the dog and those from the South China tiger [12].

Therefore, in the present study, we (i) characterized the entire mt genome sequence of *T. leonina* from the cheetah, (ii) compared it with that of *T. leonina* from the dog, and (iii) then tested the hypothesis that *T. leonina* represents a species complex.

## Methods

### Parasites and DNA isolation

All adult worms (*n* = 14; coded TC1–TC14) of *T. leonina* were obtained from a cheetah (*Acinonyx jubatus*) in Changsha Ecological Zoo, China. This cheetah was dewormed at importation to Changsha in 2012. This cheetah died in 2018 and 14 adult nematode specimens were collected by the Zoo veterinarians at necropsy. Adult nematode specimens were washed separately in physiological saline, identified preliminarily to the species level based on morphological characters [13], fixed in 70% (v/v) ethanol and stored at − 20 °C until further study was performed. Some representative examined specimens were deposited in the Specimen Museum and Resource Bank of Parasites, Lanzhou Veterinary Research Institute, Chinese Academy of Agricultural Sciences (accession no. SMRBP-LVRI-2018001-5), for retrieval and comparative study. Total genomic DNA was isolated from individual worms using sodium dodecyl sulphate/proteinase K treatment, followed by spin-column purification (Wizard^®^ SV Genomic DNA Purification System, Promega). Single specimen was further identified as *T. leonina* by PCR-based sequencing of ITS1 and ITS2 rDNA [14], and both regions had 100% nucleotide homology with those of *T. leonina* from *Lynx lynx* deposited in GenBank (GenBank accession no. JF837179). However, these sequences differed by 3% in ITS1 compared with the sequence of *T. leonina* from a wolf in China (GenBank: JF837174), and by 8% in ITS2 compared with the sequence of *T. leonina* from a golden jackal in Croatia (GenBank: MF495479).

### Long-PCR amplification and sequencing

The primers (Additional file 1: Table S1) were designed according to well-conserved regions of the mt genome sequences of *T. leonina* from the dog (GenBank: NC023504) [11]. These primers were then used to amplify the entire mt genome by long-PCR as seven overlapping fragments (2–4 kb each) from the genomic DNA. The PCR reactions contained ~ 20 ng of genomic DNA and were carried out in 50-μl reaction volumes containing 25 μl 2× Phusion Emweald Amp MAX HS PCR Master Mix (TaKaRa, Dalian, China), 2 μl of each primer, 2 μl DNA and 19 μl of ddH_2_O in a thermocycler (Bio-Rad, California, USA). Cycling amplification was executed under the following conditions: 94 °C for 2 min (initial denaturation), then 94 °C for 30 s (denaturation), 50–55 °C for 45 s (annealing), 66 °C (~ 1 kb region) for 5 min (extension) for 9 cycles, followed by 94 °C for 30 s, 50–55 °C for 45 s, and 66 °C for 5 min for 21 cycles, and a final extension at 68 °C for 10 min. Each PCR reaction yielded a single band detected in a 1.0% (w/v) agarose gel upon ethidium-bromide staining. PCR products were column-purified and then sequenced from both directions.

### Sequence analyses

Contiguous sequences were assembled manually and aligned with previously published mt genome sequence of *T. leonina* from the dog using Clustal X program v.1.83 [15] to identify gene boundaries. The open-reading frames (ORFs) and each protein-coding gene (PCG) were predicted using the program MEGA 5.0 [16], then the translation start and stop codons were identified based on comparison with those reported previously [11]. Putative secondary structures of 22 tRNA genes were identified using tRNAscan-SE [17] with manual adjustment, and two rRNA genes were predicted by comparison with those of *T. leonina* from the dog [11].

### Phylogenetic analyses

The deduced amino acid sequences of 12 PCGs were aligned individually with those of published mt genomes of other selected species of the Ascaridoidea, using *Thelazia callipaeda* as the outgroup [18]. All amino acid sequences were aligned using MAFFT 7.122 [19] and then concatenated into a single alignment. The regions and sites of ambiguous alignment were eliminated using Gblocks online server [20]. Bayesian inference (BI) was used for phylogenetic analyses as described previously [6, 11]. Phylograms were drawn using the program FigTree v.1.4.

### Sequencing of *cox*1 and analysis

The primers JB3/JB4.5 were used for PCR amplification and then sequencing of a portion of the *cox*1 gene from multiple *T. leonina* samples from the cheetah. These newly generated sequences and previously published *cox*1 sequences of *T. leonina* were aligned using the software MAFFT 7.122. Phylogenetic analysis was performed using the same methods as described above.

## Results and discussion

### General features of the mt genome of *T. leonina* from the cheetah

The entire mt genome of *T. leonina* from the cheetah is 14,685 bp in size. This mt genome contains 12 PCGs, 22 tRNA genes and two rRNA genes (Additional file 1: Table S2). The nucleotide content of the mt genome sequence of *T. leonina* from the cheetah is biased toward A+T (71.1%), in accordance with mt genomes of other nematodes sequenced to date (such as *Ascaris suum*, *Ascaris lumbricoides*, *Toxocara canis*, *Toxocara cati*) [6, 21]. All mt genes of *T. leonina* from the cheetah are transcribed in the same direction. Gene content, arrangement and composition of the mt genome of *T. leonina* from the cheetah are the same as those of other nematodes, such as *T. canis*, *T. cati* and *Toxocara malaysiensis* [21]. Additionally, the genes in the mt genome of *T. leonina* from the cheetah overlap by 1 bp between tRNA-Trp and tRNA-Glu (Additional file 1: Table S2). There are 12 intergenic regions (from 1 to 10 bp in length) in the mt genome of *T. leonina* from the cheetah (Additional file 1: Table S2). In addition, the mt genome of *T. leonina* from the cheetah contains two non-coding (AT-loop) regions (1450 bp) (Additional file 1: Table S2).

### Annotation

The mt genome of *T. leonina* from the cheetah encodes 12 PCGs. The most common start codon for the mt genome of *T. leonina* from the cheetah is TTG (7 of 12 PCGs), followed by ATT (3 of 12 PCGs), GTG (1 of 12 PCGs) and GTT (1 of 12 PCGs) (Additional file 1: Table S2). In this mt genome, the PCGs use TAG, T and TA as a stop codon (Additional file 1: Table S2), consistent with previous studies [11, 12]. Twenty-two tRNA genes in this mt genome range from 52 bp to 62 bp in length. The *rrn*L and *rrn*S genes of the mt genome of *T. leonina* from the cheetah were 959 bp and 700 bp in size, respectively (Additional file 1: Table S2), and the long and short non-coding regions were located between tRNA-Ser^UCN^ and tRNA-Asn, and *nad*4 and *cox*1, respectively (Additional file 1: Table S2).

### Comparative analyses between *T. leonina* from the cheetah and the dog

The entire mt genome of *T. leonina* from the cheetah is 375 bp and 408 bp longer than that of *T. leonina* from the dog and the South China tiger [11, 12], respectively. Nucleotide sequence difference across the entire mt genome (except for the non-coding region sequences) between *T. leonina* from the cheetah and the dog was 7.2%. This level of mt genome divergence is similar to those between different nematode species. For example, the nucleotide sequence difference was 3.8–9.4% among members of the *Pseudoterranova decipiens* species complex [22], 11.1% between *Ancylostoma duodenale* and *Ancylostoma caninum* [23, 24], 12.5% between *T. malaysiensis* and *T. cati* [21], and 6.2% between *Baylisascaris schroederi* and *Baylisascaris ailuri* [25]. For the 12 PCGs, the sequence difference between *T. leonina* from the cheetah and the dog, was 5.0–9.7% (nucleotide sequences) and 1.0–7.2% (aa sequences); these divergence levels are similar to those recorded between other congeneric nematode species. For example, the amino acid sequence divergence between *Oesophagostomum dentatum* and *O. quadrispinulatum* was 0.4–8.3% [26], and it was 0.4–7.1% between *Angiostrongylus mackerrasae* and *A. cantonensis* [27]. For the 22 tRNAs, sequence difference was 6.0% between *T. leonina* from the cheetah and the dog. For the *rrn*L and *rrn*S genes, sequence difference was both 6.1% between *T. leonina* from the cheetah and the dog (Table 1).

**Table 1:** Differences in mitochondrial nucleotides and predicted amino acids sequences between *Toxascaris leonina* from cheetah (Tc) and dog (Td)

Gene/region	Nucleotide sequence length (bp)		Nucleotide difference (%)	Number of aa		aa difference (%)
	Tc	Td	Tc/Td	Tc	Td	Tc/Td
*cox*1	1577	1577	6.3	525	525	4.4
*cox*2	697	697	5.0	232	232	1.7
*nad*3	336	336	8.3	111	111	7.2
*nad*5	1582	1582	7.4	527	527	3.6
*nad*6	435	435	8.3	144	144	4.9
*nad*4L	234	234	9.4	77	77	2.6
*nad*1	873	873	8.4	290	290	5.2
*atp*6	600	600	7.2	199	199	3.5
*nad*2	844	844	8.2	281	281	4.6
*cyt*b	1095	1095	8.3	364	364	2.7
*cox*3	768	768	7.9	255	255	1.2
*nad*4	1230	1230	9.7	409	409	1.0
*rrn*L	959	960	6.1	**–**	**–**	–
*rrn*S	700	700	6.1	–	–	–
All 22 tRNA	1250	1254	6.0	–	–	–
Non-coding	1450	1066	–	–	–	–

Nucleotide sequence difference in the mt *cox*1 gene was also assessed among *T. leonina* isolates (*n* = 23; coded TC1–TC23) from canid and felid hosts. Nucleotide differences in *cox*1 sequences among the *T. leonina* isolates from felids (*n* = 17; coded TC1–TC17) occurred at 10 sites (2.7%). Nucleotide differences among the *T. leonina* isolates from canids (*n* = 6; coded TC18-TC23) occurred at 21 sites (6.2%). However, comparison of the mt *cox*1 sequences among all the *T. leonina* isolates (*n* = 23) revealed 36 variable sites (10.6%). Our results revealed substantial sequence differences in the *cox*1 gene among different hosts, consistent with that of previous studies [8, 9].

Taken together, these results support our proposal that *T. leonina* from different hosts may present a species complex because a previous study has shown that sequence difference in mt genes between closely-related nematode species were 10–20% [28].

### Phylogenetic analysis

**Fig. 1:**
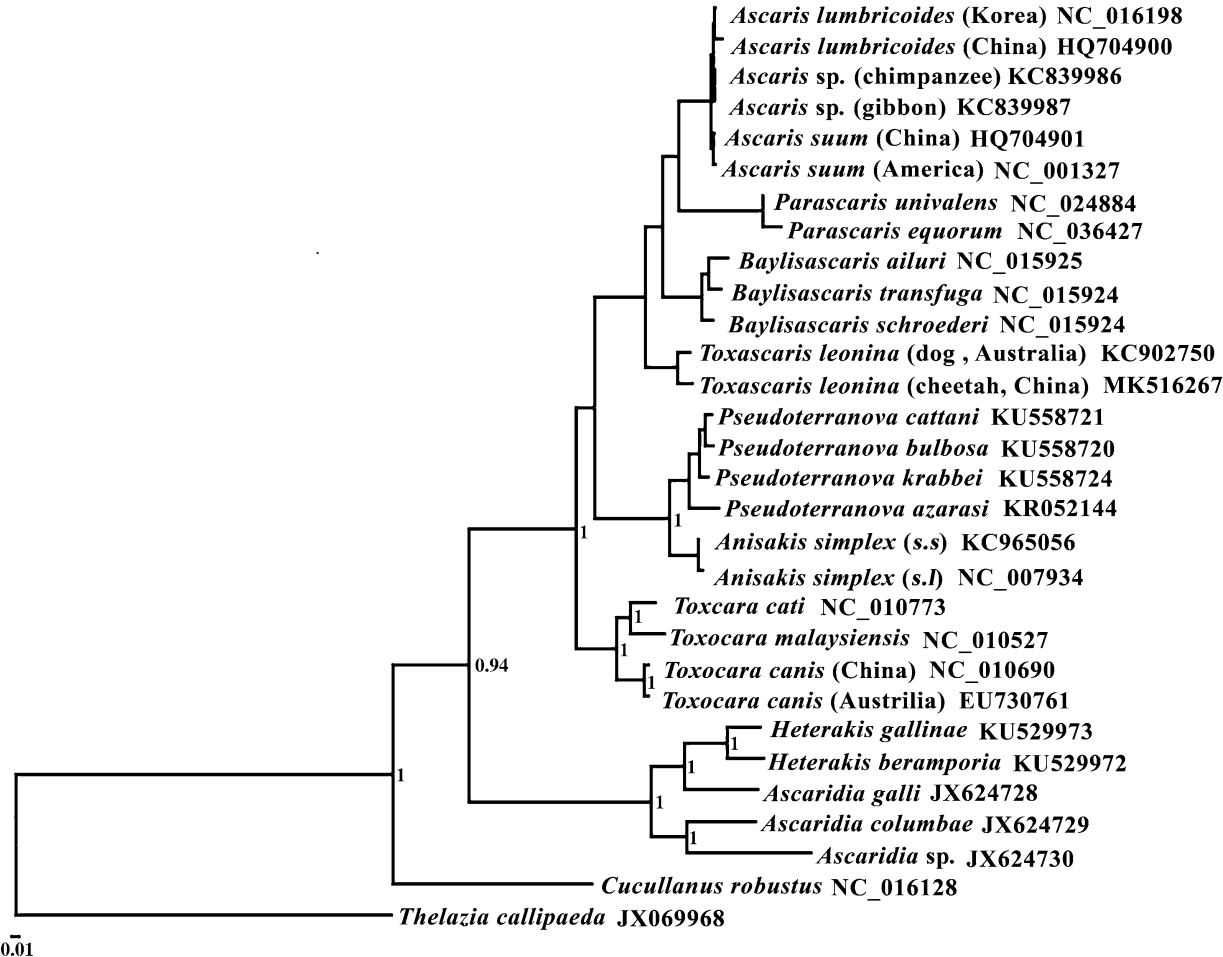
Relationships of *Toxascaris leonina* with other selected ascaridoidea nematodes based on mitochondrial sequence data. The concatenated amino acid sequences of 12 protein-coding genes were subjected to a Bayesian inference analysis using *Thelazia callipaeda* as the outgroup. Bayesian posterior probabilities values are indicated

**Fig. 2:**
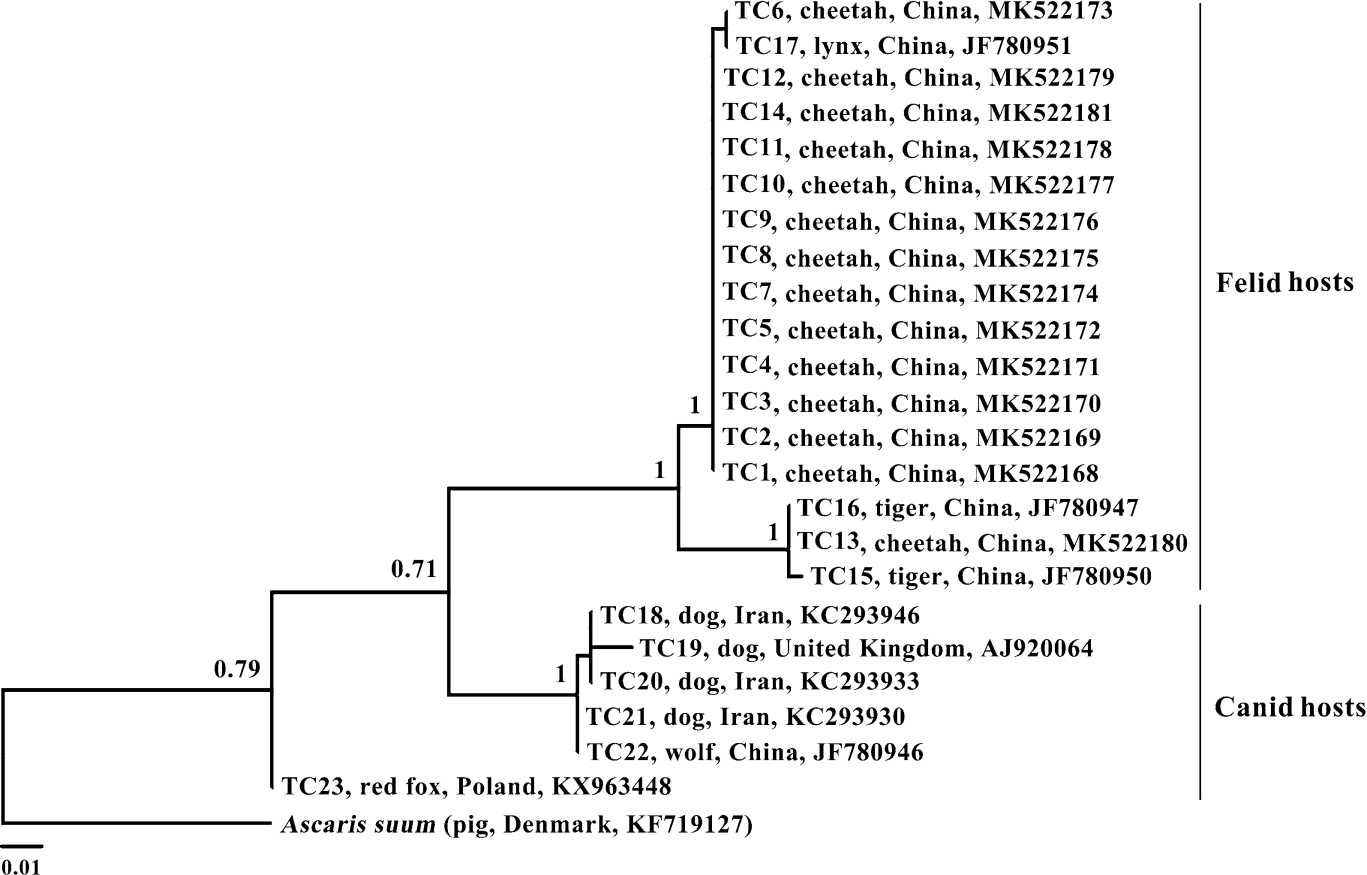
Inferred phylogenetic relationships among representative *Toxascaris leonina* samples by Bayesian inference based on mitochondrial *cox*1 nucleotide sequence data, using *Ascaris suum* as the outgroup. Bayesian posterior probabilities values are indicated

Phylogenetic analyses based on amino acid sequences of 12 PCGs showed clear genetic distinctiveness between *T. leonina* from the cheetah and the dog, and they were a sister taxon to a clade containing *Ascaris* spp., *Parascaris* spp. and *Baylisascaris* spp. of the family Ascarididae, with strong support (Bayesian posterior probabilities = 1). Basal to these taxa were the families Anisakidae, Toxocaridae, Heterakidae and Ascaridiidae (Fig. 1). The genetic distance between the two *T. leonina* isolates is approximately the same (looking at tree topologies and branch lengths) as that among the *Baylisascaris* spp. and the *Pseudoterranova* spp. (Fig. 1). In addition, phylogenetic analyses based on the mt *cox*1 sequences also showed support for the separation of *T. leonina* from different hosts into three distinct clades (Fig. 2). Our results showed that *T. leonina* isolates from felid hosts were in the same clade; however, *T. leonina* isolates from canid hosts formed two clades (Bayesian posterior probabilities > 0.71). *Toxascaris leonina* from red foxes was sister to the two remaining clades (Fig. 2). These results are consistent with a recent study [29].

## Conclusions

In summary, the present study determined the entire mt genome sequence of *T. leonina* from the cheetah. Comparative analyses of the mtDNA datasets provided genetic evidence that *T. leonina* from canid and felid hosts represents a species complex. To further test this hypothesis, additional studies are warranted (i) to explore, in detail, nucleotide variation in rDNA and mtDNA within and among *T. leonina* populations from different hosts and geographical locations; (ii) to determine the complete mt genomes of *T. leonina* from other hosts; and (iii) to undertake detailed morphological studies of *T. leonina* from a range of hosts.

## Additional file

**Additional file 1: Table S1.** Sequences of primers used to amplify Long-PCR fragments from *Toxascaris leonina.* **Table S2.** Mitochondrial genome organization of *Toxascaris leonina* from cheetah (Tc) and *Toxascaris leonina* from dog (Td).

## Abbreviations

mt: mitochondrial
mtDNA: mitochondrial DNA
rDNA: ribosomal DNA
BI: Bayesian inference
*nad*2: NADH dehydrogenase subunit 2
*cox*1: cytochrome *c* oxidase subunit 1
*cox*2: cytochrome *c* oxidase subunit 2
ATP6: ATP synthase F0 subunit 6
*cox*3: cytochrome *c* oxidase subunit 3
*nad*3: NADH dehydrogenase subunit 3
*nad*5: NADH dehydrogenase subunit 5
*nad*4: NADH dehydrogenase subunit 4
*nad*4L: NADH dehydrogenase subunit 4L
*nad*6: NADH dehydrogenase subunit 6
*cyt*b: cytochrome b
*nad*1: NADH dehydrogenase subunit 3
tRNA: transfer RNA
*rrn*L: large subunit of rRNA
*rrn*S: small subunit of rRNA
ITS: internal transcribed spacers
BI: Bayesian inference
