# Supplementary material for: Comparative analysis of mitochondrial DNA datasets indicates that Toxascaris leonina represents a species complex
Source: Parasit Vectors. 2019 May 2;12:194. doi: 10.1186/s13071-019-3447-2 (PMC6498696; doi:10.1186/s13071-019-3447-2)
Supplement: Supplementary file 1 — Additional file 1: Table S1. Sequences of primers used to amplify Long-PCR fragments from Toxascaris leonina. Table S2. Mitochondrial genome organization of Toxascaris leonina from cheetah (Tc) and Toxascaris leonina from dog (Td). [file 13071_2019_3447_MOESM1_ESM.docx]

**Additional file 1: Table S1** Sequences of primers used to amplify Long-PCR fragments from *Toxascaris leonina*

| Primer | Sequence (5’ to 3’) | Amplified region |
| --- | --- | --- |
| TC1F | TTGGGTTGATTGGTTGTGTTG | Partial *cox*1-C-M-D-G-*cox*2-H-partial *rrn*L  Partial *rrn*L-*nad*3-partial *nad*5  Partial *nad*5-A-P-V- *nad*6-*nad*4L-W-E-partial *rrn*S  Partial *rrn*S-S_2_-NCL-N-Y-partial *nad*1  Partial *nad*1-*atp*6-K-L_2_-S_1_-partial *nad*2  Partial *nad*2-I-R-Q-F-*cytb*-L_1_- partial *cox*3  Partial *cox*3-T-*nad*4-NCR-partial *cox*1 |
| TC1R | GATTTTCTGTTAACTCCGAAGTAAT |  |
| TC2F | TTGTGCTTTTAATGGGTTCTGG |  |
| TC2R | CCTTTTTCAAATCCTCCTCCAC |  |
| TC3F | AGGAGGATTTGAAAAAGGTGGT |  |
| TC3R | GTAAAAGTTGCTAATGAGGGCT |  |
| TC4F | GAGAGCCCTCATTAGCAACT |  |
| TC4R | AGCAATCTCATAAGACACACTC |  |
| TC5F | TTGAAGAAGGAGCAGATGAC |  |
| TC5R | AAGAAACCCTAAAAGGAACA |  |
| TC6F | TGGTGATTTCTTCTACGGAGT |  |
| TC6R | CAAAAGCCTGTAATGAGCCC |  |
| TC7F | GGTGGGGTTTGGTCTCCTAT |  |
| TC7R | GCACGAGAATCCAAATCCAT |  |

**Additional file 1: Table S2.** Mitochondrial genome organization of *Toxascaris leonina* from cheetah (Tc) and *Toxascaris leonina* from dog (Td)

| Gene/region | Position and nt size (bp) | | Ini/Ter codon | | Intergenic nucleotides | |
| --- | --- | --- | --- | --- | --- | --- |
|  | Tc | Td | Tc | Td | Tc | Td |
| *cox1* | 1-1577(1577) | 1-1577(1577) | TTG/TA | TTG/TA | 0 | 0 |
| tRNA-Cys (C) | 1578-1633(56) | 1578-1633(56) |  |  | 0 | 0 |
| tRNA-Met (M) | 1640-1700(61) | 1641–1701(61) |  |  | +6 | +7 |
| tRNA-Asp (D) | 1701-1757(57) | 1702–1758(57) |  |  | 0 | 0 |
| tRNA-Gly (G) | 1761-1816(56) | 1762–1818(57) |  |  | +3 | +3 |
| *cox2* | 1817-2513(697) | 1819–2515(697) | TTG/T | TTG/T | 0 | 0 |
| tRNA-His (H) | 2514-2572(59) | 2516-2574(59) |  |  | 0 | 0 |
| *rrnL* | 2573- 3531(959) | 2575-3534(960) |  |  | 0 | 0 |
| *nad3* | 3533-3868(336) | 3536-3871(336) | TTG/TAG | TTG/TAG | +1 | +1 |
| *nad5* | 3869-5450(1582) | 3872-5453(1582) | ATT/T | ATT/T | 0 | 0 |
| tRNA-Ala (A) | 5451-5506(56) | 5454-5509(56) |  |  | 0 | 0 |
| tRNA-Pro (P) | 5507-5561(55) | 5510-5564(55) |  |  | 0 | 0 |
| tRNA-Val (V) | 5562-5617(56) | 5565-5620(56) |  |  | 0 | 0 |
| *nad6* | 5618-6052(435) | 5621-6055(435) | TTG/TAG | TTG/TAG | 0 | 0 |
| *nad4L* | 6054-6287(234) | 6056-6289(234) | ATT/TAG | ATT/TAG | 0 | 0 |
| tRNA-Trp (W) | 6288-6344(57) | 6290-6346(57) |  |  | 0 | 0 |
| tRNA-Glu (E) | 6344–6398(55) | 6347-6405(59) |  |  | -1 | 0 |
| *rrnS* | 6399-7098(700) | 6406-7105(700) |  |  | 0 | 0 |
| tRNA-Ser^UCN^ (S2) | 7109-7162(54) | 7116–7169(54) |  |  | +10 | +10 |
| Non-codin region (NCR) | 7163-8498(1336) | 7170–8123(954) |  |  | 0 | 0 |
| tRNA-Asn (N) | 8499-8556(58) | 8124-8181(58) |  |  | 0 | 0 |
| tRNA-Tyr (Y) | 8557-8613(57) | 8182-8239(58) |  |  | 0 | 0 |
| *nad1* | 8614-9486(873) | 8240-9112(873) | TTG/TAG | TTG/TAG | 0 | 0 |
| *atp6* | 9494-10093(600) | 9120-9719(600) | ATT/TAG | ATT/TAG | +7 | +7 |
| tRNA-Lys (K) | 10100-10161(62) | 9727-9787(61) |  |  | +6 | +7 |
| tRNA-Leu^UUR^ (L2) | 10167-10221(55) | 9793-9847(55) |  |  | +5 | +5 |
| tRNA-Ser^AGN^ (S1) | 10222-10273(52) | 9848-9899(52) |  |  | 0 | 0 |
| *nad2* | 10274-11117(844) | 9900-10,743(844) | GTG/T | GTG/T | 0 | 0 |
| tRNA-Ile (I) | 11118-11178(61) | 10,744-10,804(61) |  |  | 0 | 0 |
| tRNA-Arg (R) | 11179-11233(55) | 10,805-10,859(55) |  |  | 0 | 0 |
| tRNA-Gln (Q) | 11235-11289(55) | 10,861-10,915(55) |  |  | +1 | +1 |
| tRNA-Phe (F) | 11293-11352(60) | 10,919-10,978(60) |  |  | +3 | +3 |
| *cytb* | 11362-12456(1095) | 10,988-12,082(1095) | TTG/TAG | TTG/TAA | +9 | +9 |
| tRNA-Leu^CUN^ (L1) | 12458-12515(58) | 12,086-12,141(56) |  |  | +1 | +3 |
| *cox3* | 12516-13283(768) | 12,144-12,911(768) | GTT/TAG | GTT/TAG | 0 | +2 |
| tRNA-Thr (T) | 13285-13340(56) | 12,913-12,968(56) |  |  | +1 | +1 |
| *nad4* | 13341-14570(1230) | 12,969-14,198(1230) | TTG/TAG | TTG/TAG | 0 | 0 |
| Non-coding region (NCR) | 14571-14685(114) | 14,199-14,310(112) |  |  | 0 | 0 |
